# GDF11 induces mild hepatic fibrosis independent of metabolic health

**DOI:** 10.18632/aging.104182

**Published:** 2020-10-28

**Authors:** Jan Frohlich, Kristina Kovacovicova, Tommaso Mazza, Maria R. Emma, Daniela Cabibi, Michelangelo Foti, Cyril Sobolewski, Jude A. Oben, Marion Peyrou, Francesc Villarroya, Maurizio Soresi, Rita Rezzani, Melchiorre Cervello, Francesca Bonomini, Anna Alisi, Manlio Vinciguerra

**Affiliations:** 1 International Clinical Research Center, St. Anne's University Hospital, Brno, Czech Republic; 2 Bioinformatics Unit, IRCCS Casa Sollievo della Sofferenza, San Giovanni Rotondo, Italy; 3 Institute for Biomedical Research and Innovation, National Research Council (CNR), Palermo, Italy; 4 Department of Health Promotion Sciences, Maternal and Infantile Care, Internal Medicine and Medical Specialties, University of Palermo, Palermo, Italy; 5 Department of Cell Physiology and Metabolism, Faculty of Medicine, University of Geneva, Geneva, Switzerland; 6 Institute for Liver and Digestive Health, Division of Medicine, University College London (UCL), London, United Kingdom; 7 Department of Biochemistry and Molecular Biomedicine, Institute of Biomedicine of the University of Barcelona, Barcelona, Catalonia, Spain; 8 Institut de Recerca Hospital de la Santa Creu i Sant Pau, Barcelona, Catalonia, Spain; 9 CIBER Fisiopatología de la Obesidad y Nutrición, Barcelona, Catalonia, Spain; 10 Anatomy and Physiopathology Division, Department of Clinical and Experimental Sciences, University of Brescia, Brescia, Italy; 11 Interdepartmental University Center of Research “Adaption and Regeneration of Tissues and Organs-(ARTO)”, University of Brescia, Brescia, Italy; 12 Research Area for Multifactorial Diseases, Research Unit of Molecular Genetics of Complex Phenotypes, Bambino Gesù Children's Hospital, IRCCS, Rome, Italy

**Keywords:** liver, NAFLD, NASH, fibrosis, growth differentiation factor 11

## Abstract

Background & aims: Growth Differentiation Factor 11 (GDF11) is an anti-aging factor, yet its role in liver diseases is not established. We evaluated the role of GDF11 in healthy conditions and in the transition from non-alcoholic fatty liver disease (NAFLD) to non-alcoholic steatohepatitis (NASH).

Results: GDF11 mRNA levels positively correlated with NAFLD activity score and with CPT1, SREBP, PPARγ and Col1A1 mRNA levels, and associated to portal fibrosis, in morbidly obese patients with NAFLD/NASH. GDF11-treated mice showed mildly exacerbated hepatic collagen deposition, accompanied by weight loss and without changes in liver steatosis or inflammation. GDF11 triggered ALK5-dependent SMAD2/3 nuclear translocation and the pro-fibrogenic activation of HSC.

Conclusions: GDF11 supplementation promotes mild liver fibrosis. Even considering its beneficial metabolic effects, caution should be taken when considering therapeutics that regulate GDF11.

Methods: We analyzed liver biopsies from a cohort of 33 morbidly obese adults with NAFLD/NASH. We determined the correlations in mRNA expression levels between GDF11 and genes involved in NAFLD-to-NASH progression and with pathological features. We also exposed wild type or obese mice with NAFLD to recombinant GDF11 by daily intra-peritoneal injection and monitor the hepatic pathological changes. Finally, we analyzed GDF11-activated signaling pathways in hepatic stellate cells (HSC).

## INTRODUCTION

Liver fibrosis is a wound healing response that is activated during various types of liver injury. If uncontrolled, liver fibrosis can progress into liver cirrhosis and eventually into hepatocellular carcinoma (HCC) [1, 2]. HCC ranks consistently among the top five most common cancers and leading causes of cancer-associated deaths worldwide [3]. Liver fibrosis is one of the major risk factors for HCC development, with up to 90% of patients displaying cirrhosis at the time of diagnosis [4]. The most common causes of liver fibrosis in industrialized countries are alcohol abuse, viral infections (HBV, HCV), and obesity-related non-alcoholic fatty liver disease (NAFLD) and non-alcoholic steatohepatitis (NASH). NAFLD is characterized by increased intrahepatic fat accumulation because of the excessive dietary fat and carbohydrate intake and lower rate of fatty acid oxidation. NAFLD is the most common liver disorder in the Western world, affecting up to 30% of the general adult population [5]. Up to 15% of NAFLD cases will progress to NASH and to fibrosis. Identifying molecular targets for treating NAFLD, or for avoiding its fibrotic exacerbation, has become a major public health concern.

At the mechanistic level, persistent injury and damage cause hepatocytes to release reactive oxygen species (ROS) and other mediators that activate parenchymal hepatic stellate cells (HSCs). These cells perpetuate liver fibrosis by ultimately promoting collagen production and ECM deposition. Transforming growth factor-beta (TGF-β) is one of the most potent HSC activators and markedly stimulates the synthesis of extracellular matrix (ECM) components and remodeling factors [including collagens I and III, matrix metalloproteinases (MMPs) and metalloproteinase inhibitors (TIMPs)] [6, 7].

Growth differentiation factor 11 (GDF11) belongs to the TGF-β superfamily. Similar to other TGF-β superfamily members, GDF11 signaling is transmitted via type I and II serine/threonine kinase receptors (8). In particular, GDF11 binds to activin II receptors (ACTRIIB), resulting in the subsequent recruitment of the activin receptor-like kinases (ALKs) ALK4, ALK5 and ALK7. Activated receptor complexes transduce the signal via Smad2/3 complex phosphorylation, and this complex is translocated into the cell nucleus where it regulates gene expression [8, 9]. In the context of liver diseases, exogenous GDF11 expression correlates with tumor suppression in HCC cells *in vitro* [10, 11], but worsens hepatocellular injury and liver regeneration after liver ischemia reperfusion injury *in vivo* [12]. If GDF11 administration might prevent high fat diet (HFD)-induced NAFLD and obesity in mice, is a matter of debate: Walker RG et al. showed that exogenous delivery of recombinant GDF11 has no effect on NAFLD [13]. Conversely, others have reported that GDF11 impairs the development of HFD-induced NAFLD and the transition to liver fibrosis: in these studies, GDF11 was delivered by hydrodynamic injection-mediated gene transfer [14], or by adenoassociated virus vectors (AAV) [15], respectively.

GDF11 is expressed in various tissues and has an important role throughout the mammalian embryonic development, where it affects spinal cord anterior/posterior patterning, the development of urogenital system, stomach, spleen, endocrine pancreas, and olfactory neurogenesis [8]. Over the past decade, seminal studies have shown that systemic restoration of GDF11 levels reverses age-related phenotypes in aged mice, rejuvenates heart and skeletal muscle [16, 17] and improves the vascular and neurogenic rejuvenation in the brain [18, 19]. As such, GDF11 has been heralded as a powerful “anti-aging” factor. Conflicting reports, however, have queried whether circulating GDF11 levels decrease with age [20, 21]. Furthermore, supraphysiological levels could lead to cachexia [22].

These controversial data highlight that the true local and systemic effects of GDF11 in health and disease are not fully delineated. The aim of the present study was to specifically characterize the potential role of GDF11 in liver fibrosis, including the progression of NAFLD to NASH. We investigated the potential impact of GDF11 in a cohort of patients with NAFLD/NASH, in murine models and *in vitro*.

## RESULTS

### GDF11 mRNA levels correlate positively with the severity of human NAFLD

**Figure 1:**
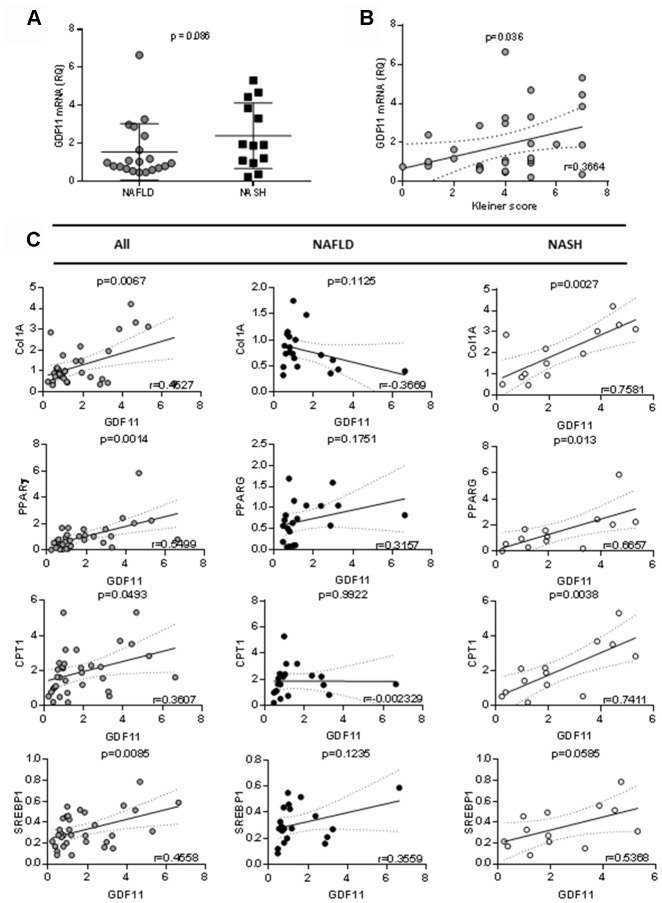
**Correlation between GDF11 mRNA levels, clinico-pathologic characteristics and gene expression in morbidly obese patients (n=33).** Correlations between GDF11 mRNA levels and (**A**) NAFLD or NASH, (**B**) Kleiner score (0-8) and (**C**) expression levels of Col1A1, SREBP1, PPARγ and CPT1 in the whole cohort of morbidly obese patients (n=33) and subgroups with NAFLD (n=20) or NASH (n=13). The Pearson correlation’s coefficient is shown.

**Figure 2:**
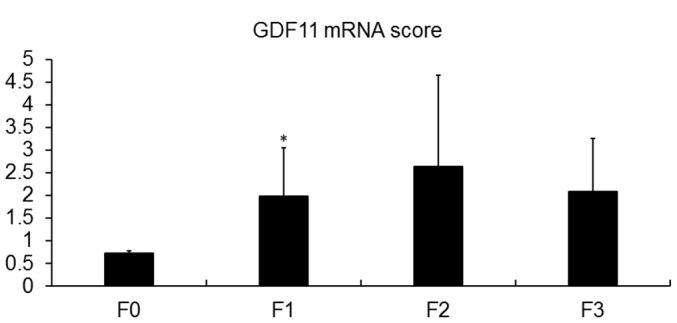
**Association of GDF11 expression with liver fibrosis.** GDF11 mRNA levels in morbidly obese patients (n=33), according to their fibrosis score, determined by histological assessment (F0-F4). * p<0.05 versus F0 (T-test).

We analyzed the hepatic mRNA expression of GDF11 gene in a cohort of 33 obese patients (BMI 45.02 ± 6.41) with NAFLD (Supplementary Table 1) in comparison with pooled liver samples from 9 healthy donors. GDF11 mRNA levels tended to increase with NAFLD to NASH progression (p=0.086) (Figure 1A) and correlated positively with NAS score (0-8) (p=0.036) (Figure 1B, Supplementary Table 2). We found no differences between GDF11 mRNA levels and Type 2 diabetes in obese patients with either NASH or NAFLD (Supplementary Figure 1). However, GDF11 mRNA correlated negatively with blood glucose levels in NAFLD, but not in NASH patients (Supplementary Table 3). We also measured the hepatic expression values of a panel of genes involved in NAFLD progression (FASN, SREBP1, PPARα, PPARγ, CPT1, Col1A1) in all patients (n=33). Significant positive correlations were found between the mRNA levels of GDF11 *vs* PPARγ (p<0.01), CPT1 (p<0.05), SREBP1 (p<0.01) and Col1A1 (p<0.01) (Figure 1C), but not between GDF11 and FASN or PPARα (Table 1). When we separately analyzed the subjects with NAFLD (n=20) or NASH (n=13), the positive correlations between GDF11 *vs* PPARγ, CPT1 and Col1A1 mRNA levels were maintained and reinforced in the NASH group, but were no longer present in the NAFLD group (Figure 1C). Furthermore, GDF11 mRNA levels tended to correlate with progression of liver fibrosis stages (F0 to F4) in our cohort. In particular, a significant increase in GDF11 mRNA was observed in F1 (portal fibrosis) compared to F0 (no fibrosis) (Figure 2). Altogether, these findings demonstrate a positive correlation between increased GDF11 expression and worsened NAFLD to NASH progression, suggesting a pathogenic and fibrogenic role of this factor.

**Table 1:** Pearson’s correlation between mRNA levels of GDF11 and genes involved in lipid metabolism in our cohort of obese patients.

**ALL**	**GDF11. vs. Col1A**	**GDF11. vs. FASN**	**GDF11. vs. PPARG**	**GDF11. vs. CPT1**	**GDF11. vs. SREBP1**	**GDF11. vs. PPARa**
Pearson r	0.456	0.04332	0.527	0.3398	0.444	0.3189
P value	0.0067	0.8078	0.0014	0.0493	0.0085	0.066
NAFLD	GDF11. vs. Col1A	GDF11. vs. FASN	GDF11. vs. PPARG	GDF11. vs. CPT1	GDF11. vs. SREBP1	GDF11. vs. PPARa
Pearson r	-0.366	-0.2601	0.3157	-0.0023	0.3559	0.3276
P value	0.1125	0.2682	0.1751	0.9922	0.1235	0.1585
NASH	GDF11. vs. Col1A	GDF11. vs. FASN	GDF11. vs. PPARG	GDF11. vs. CPT1	GDF11. vs. SREBP1	GDF11. vs. PPARa
Pearson r	0.7581	0.3356	0.6657	0.7411	0.5368	0.4897
P value	0.0027	0.2623	0.013	0.0038	0.0585	0.0894

Analysis was carried out in all patients (n=33) or they were divided into two subgroups according to the presence NAFLD (n=20) or NASH (n=13).

### GDF 11 activates HSC in wild type mice

**Figure 3:**
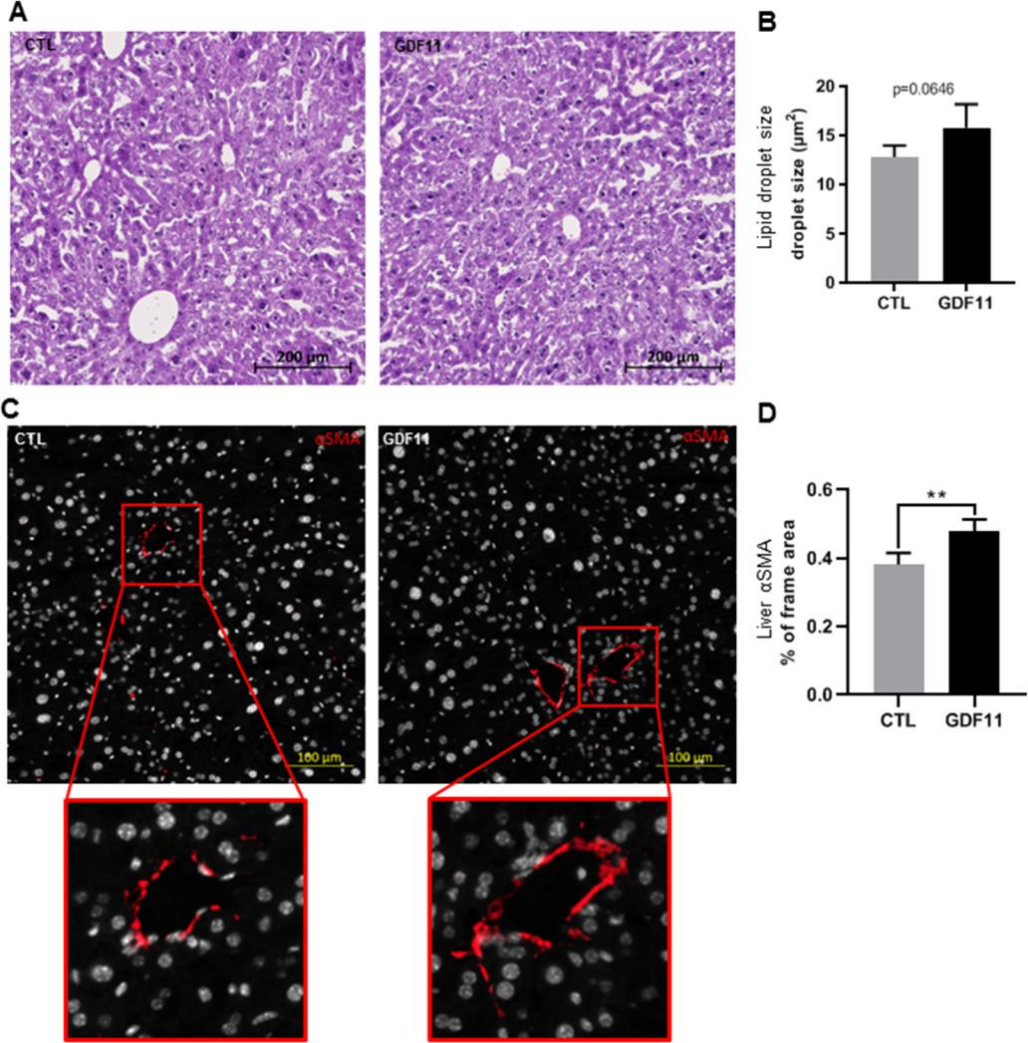
**GDF11 induces HSCs activation in mice. 16-18 months old wild type C57/BL6J mice (n=5 mice per group) were injected daily (for nine days) with either GDF11 (1 mg/kg) or saline.** (**A**) Representative images of H&E stained livers (200x magnification) from the livers of CTL and GDF11-treated mice. (**B**) Quantitative morphometric analysis of total lipid area (%) as in (**A**). (**C**) Representative images of αSMA immunostaining (200x magnification) in the livers of CTL and GDF11-treated mice. (**D**) Quantitative morphometric analysis of αSMA immunostaining (% of total imaged area, n=5 per group, at least fifteen randomly chosen fields per sample were evaluated). Data are represented as the mean ± SD. ** p<0.01 (Mann-Whitney U-test).

As GDF11 correlated positively with the severity of human NAFLD/NASH, we sought to test the effects of GDF11 on NAFLD progression *in vivo*. It has been recently reported that systemic GDF11 administration to wild type mice triggers a caloric restriction-like phenotype, with weight loss and without affecting appetite or physical activity [23]. Here we analyzed the hepatic phenotype at the histological level in these mice. Nine-day consecutive treatment with recombinant GDF11, injected IP at 1 mg/kg, in 16-18 months old wild type mice did not induce overt lipid accumulation or NAFLD, as quantified in micro steatosis or total fat accumulation (measured as % of the total imaged area) on H&E stained liver sections (Figure 3A, 3B) (n=5 per group). Conversely, recombinant GDF11 significantly increased the number of activated HSCs cells in perivenular areas, as evidenced by specific αSMA immunostaining, compared to the control (CTL) group (Figure 3C, 3D). These data suggest that GDF11 might have mild pro-fibrogenic effects *in vivo*, regardless of its effects on lipid content.

### GDF 11 aggravates fibrosis in ob/ob mice

**Figure 4:**
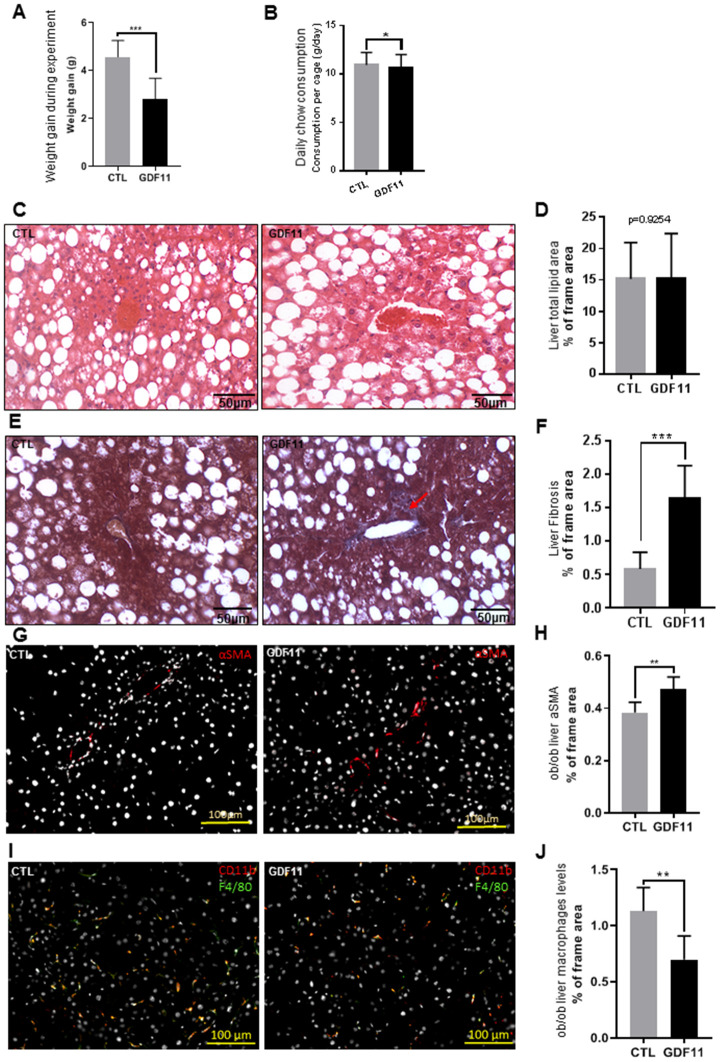
**GDF11 accelerates NAFLD progression in obese mice. Obese (*ob/ob*) mice (n=12 mice per group) were injected daily (for 14 days) with either GDF11 (0.1 mg/kg) or saline.** Graphs showing (**A**) average weight gain during the experiment and (**B**) average food consumption per cage (n=2 mice per cage). (**C**) Representative images of H&E stained livers from CTL and GDF11-treated *ob/ob* mice (n=6 mice per group, 200x magnification). (**D**) Quantitative morphometric analyses of total lipid area (% of imaged area) as in (**C**) (n=6 per group, at least fifteen randomly chosen fields per sample were evaluated). (**E**) Representative images of Masson’s trichrome histological staining to visualize liver fibrosis in CTL and GDF11-treated *ob/ob* mice (n=6 per group, at least fifteen randomly chosen fields per animal were evaluated, 200x magnification). The red arrow indicates area with increased staining. (**F**) Morphometric quantification of liver fibrosis (% of total imaged area) (n=6 per group, at least fifteen randomly chosen fields per sample were evaluated). (**G**) Representative images of αSMA immunostained livers from CTL and GDF11-treated *ob/ob* mice (n=6 per group, 200x magnification). (**H**) Quantitative morphometric analyses of total αSMA stained area (%) as in (**G**) (n=6 per group). (**I**) Representative images of F4/80 immunostained livers from CTL and GDF11-treated *ob/ob* mice (n=6 per group, 200x magnification). (**J**) Quantitative morphometric analyses of total F4/80 stained area (%) as in (**I**) (n=6 per group at least fifteen randomly chosen fields per sample were evaluated). Data are represented as the mean±SD (or indicated otherwise) * p<0.05, ** p<0.01, *** p<0.001 (Mann-Whitney U-test).

Next, we attempted to address the question whether GDF11 accelerates the progression from NAFLD to NASH, a major clinical issue. To this purpose, we used the *ob/ob* mouse model of obesity-dependent NAFLD. Two groups of *ob/ob* mice (n=12 per cohort) were injected daily i.p. for 14 days with either saline (CTL) or GDF11 (Supplementary Figure 2). The total weight gain of mice during the experiment was significantly lower (p<0.001) in the GDF11-treated group (2.6±0.9 g) than in the CTL group (4.5±0.7 g) (Figure 4A). Daily chow consumption per cage was slightly lower in the GDF11-treated group (10.69±1.3 g) when compared to the CTL group (10.95±1.3 g) (Figure 4B). We found no differences in the weights of organs or tissues (liver, heart, quadriceps muscle and abdominal fat) between the two groups (Supplementary Figure 3A, 3B).

**Figure 5:**
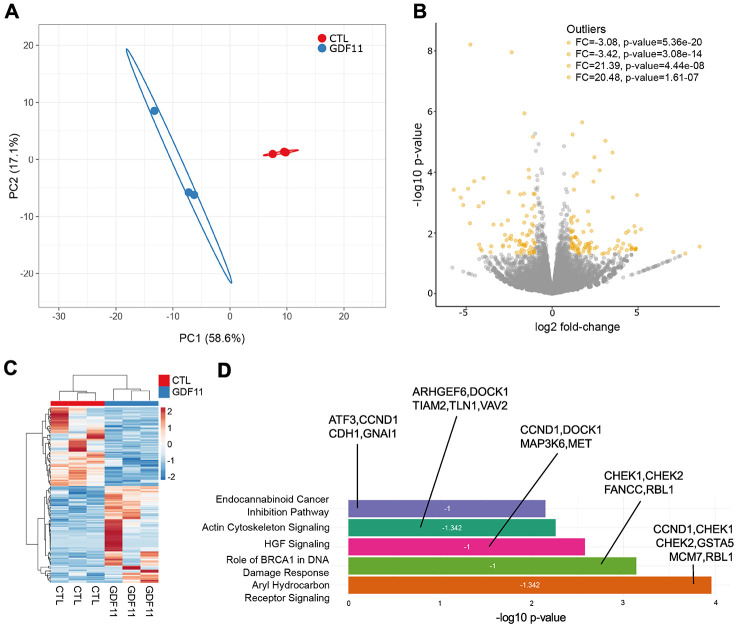
**Anti-fibrogenic pathways are inhibited after GDF11 treatment in the liver of *ob/ob* mice.** (**A**) Principal Component Analysis applied to expression profiles of 179 genes in control and GDF11-treated livers from *ob/ob* mice (n=3 per group). (**B**) Volcano plot displaying differences in gene expression (|log_2_ fold-change| >2, -log_10_ p-value >1.3). The top 4 outliers, not in the plot, are evidenced in terms of fold change (FC) and significance (p-value). (**C**) Heatmap showing differences in mRNA expression levels between control and GDF11-treated *ob/ob* mice (n=3 per group). (**D**) Bar plot reporting biological pathways in crescent statistical significance order (bar length), together with the functional inhibition score of the pathways (z-score value within the bar) and the differentially expressed genes representing each pathway.

At the hepatic level, we performed a histological assessment of the liver parenchymal architecture by H&E staining but did not identify any differences in micro/macrosteatosis or total fat accumulation (measured as % of the total imaged area) between CTL and GDF11-treated *ob/ob* mice (Figure 4C, 4D). We then performed Masson’s trichrome staining to evaluate liver fibrosis. GDF11-treated *ob/ob* mice displayed significantly more perivenular liver fibrosis (1.64%±48 of total imaged liver area) compared to CTL mice (0.58%±25) (p<0.001) (Figure 4E, 4F). Consistently, the number of positive cells for αSMA, a phenotypic marker of activated HSC, was significantly increased in the liver GDF11-treated *ob/ob* mice (Figure 4G, 4H). The transition from mild form of NAFLD to NASH depends on the progression of liver inflammation. Liver macrophages are a population consisting of tissue-resident macrophages or Kupffer cells (KC) and bone marrow infiltrated macrophages, involved in the early phase and progression of NASH, respectively, by producing pro-inflammatory cytokines and supporting HSC activation. Surprisingly, the number of positive cells for F4/80, a phenotypic marker of both KC and macrophages, was significantly reduced in the liver GDF11-treated *ob/ob* mice (Figure 4I, 4J). Moreover, increased fibrosis upon GDF11 administration was not accompanied by increased liver injury as detected using the TUNEL assay (Supplementary Figure 4). To unravel the gene expression patterns and related signaling pathways that might be responsible for the observed fibrotic changes upon GDF11 treatment, we analyzed whole livers from control and GDF11-treated *ob/ob* animals by RNA-Seq (n=3 per group). The two groups clustered distinctively by PCA (Figure 5A). Treatment with GDF11 had a substantial effect on the overall gene expression profile (Figure 5B, 5C). In total, we identified 179 differentially expressed genes, of which 74 were significantly over-expressed (FC ≥ 2) and 105 were downregulated (FC ≤ -2) (Supplementary File 1).

To determine the biological pathways significantly represented by these differentially expressed genes, we used the Ingenuity Pathway Analysis (IPA) software package. IPA uncovered significant negative association between GDF11 treatment and the activation of aryl hydrocarbon receptor (AHR) signaling pathway (z-score= -1.342), BRCA1-dependent DNA damage response (z-score= -1) and HGF signaling (z-score= -1) pathways (Figure 5D). These pathways were the top three most significantly deregulated canonical pathways/cell processes (Figure 5D), and their inhibition has been consistently associated to the development of liver fibrosis and disease [24–26]. Altogether, these findings demonstrate that GDF11 administration triggers the development of liver fibrosis in an obesity-dependent NAFLD background.

### GDF11 activates hepatic stellate cells

**Figure 6:**
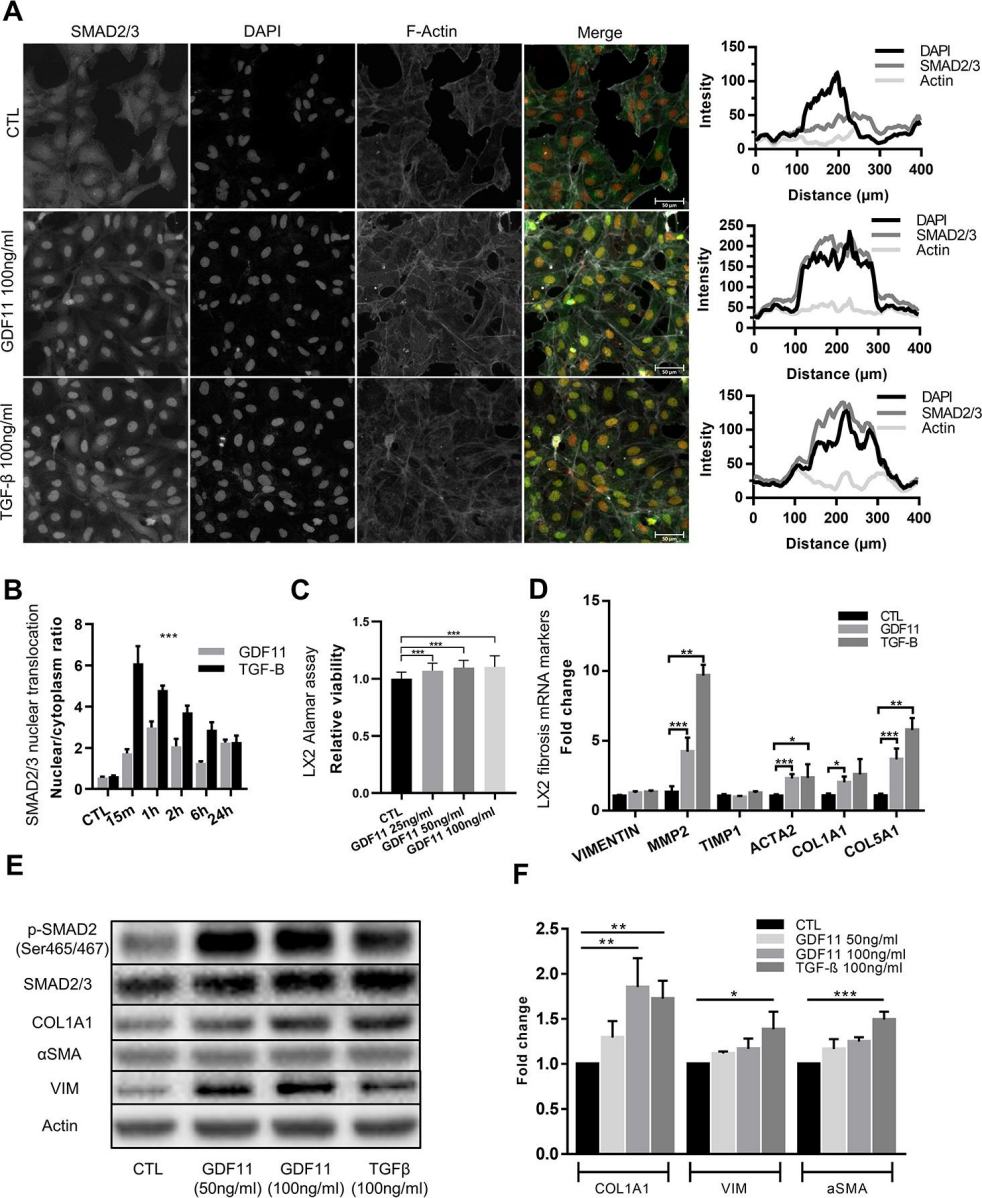
**Stellate cells are activated and produce ECM components after GDF11 exposure.** (**A**) Activation of the LX2 stellate cell line by GDF11 (100 ng/ml) or TGF-β (100 ng/ml, positive control) (scale= 100μm). (**B**) The nuclear translocation ratio of SMAD2/3 complexes after GDF11 and TGF-β treatment (n=3 per group). (**C**) Cell viability was assessed in LX2 cells, treated with different doses of GDF11 (25, 50, 100 ng/ml) for 48 hours, by using alamarBlue™ Cell Viability Reagent. (**D**) Relative mRNA expression of liver fibrosis/stellate cells activation markers in LX2 cells after exposure to GDF11 (100 ng/ml) or TGF-β (100 ng/ml). (**E**) Protein expression levels of liver fibrosis/stellate cells activation markers in LX2 cells after GDF11 or TGF-β exposure. Protein expression of selected effectors (pSMAD2, COL1A1, αSMA, vimentin (VIM), actin, GAPDH) were quantitatively assessed by immunoblotting in LX2 cells exposed or not for 48 h to GDF11 (50 or 100 ng/ml) or stimulated for 48h with TGF-β (100 ng/ml). Images are representative of three independent experiments. (**F**) Data quantification represents the means ± SD. * p<0.05; ** p<0.01; *** p<0.001 (Mann-Whitney U-test).

Hepatic stellate cells (HSCs) are the major contributors to ECM deposition in the liver. Upon activation, HSCs start producing ECM components, including different types of collagens, fibronectin, MMPs and their inhibitors (6). We thus tested the ability of GDF11 to activate human LX2 hepatic stellate cells *in vitro*. We found that GDF11 could trigger SMAD2/3 nuclear translocation (Figure 6A), with the highest response observed 1h after GDF11 incubation (Figure 6B). As a positive control we employed TGF-β - a well-known HSC activator. SMAD2/3 nuclear translocation was faster (peaked at 15 min) and more accentuated upon TGF-β treatment, compared to GDF11 (Figure 6A and 6B). Interestingly, increasing doses of GDF11 (25, 50, 100 ng/ml) lead to slightly increased LX2 cell proliferation (Figure 6C). Also, incubating LX2 cells with either GDF11 or TGF-β for 24 h led to significantly elevated mRNA levels of MMP2, αSMA/ACTA2, Col1A1 and Col5A1 — markers for stellate cell activation and liver fibrosis (Figure 6D). Expression levels of these genes were, however, more elevated upon TGF-β treatment compared to GDF11, consistent with SMAD2/3 translocation (Figure 6D). Inhibiting ALK5 with Repsox strongly attenuated GDF11-mediated pro-fibrogenic gene expression in LX2 cells, with the exception of a paradoxical increase in α-SMA/ACTA2 mRNA levels (Figure 6D). GDF11 also significantly increased Col1A1, and tended to increase vimentin and α-SMA, at the protein level, in LX2 cells similar to TGF-β treatment (Figure 6E, 6F). Taken together, these data indicate that GDF11 has TGF-β like, ALK5-dependent pro-fibrogenic effects *in vitro*.

## DISCUSSION

GDF11 belongs to the TGF-β superfamily, and has fundamental biological functions in controlling anterior-posterior patterning by regulating the expression of Hox genes [27, 28]. In 2015, Egerman et al. reported that GDF11 circulating levels increase during aging [20], which is a major risk factor for the exacerbation of liver disease progression [29]. In the current work, we thus studied the role of GDF11 during the progression from NAFLD to NASH *in vivo*, which is poorly understood. This progression is the main risk factor for the insurgence of HCC [3, 30, 31]. Our data suggest that GDF11 supplementation impinges on the ALK5/SMAD-dependent signaling pathway in HSC cells, in a way that could be similar or overlapping with the TGF-β and other pro-fibrogenic signaling pathways. However, the potential cross-talk between GDF11 (and/or other GDFs) and TGF-β signaling pathways, despite activating common SMAD downstream proteins, remains to be addressed.

In 2014, GDF11 received large media attention as it was described as a potential life extension factor, based on the results of heterochronic (young/old) parabiosis experiments performed in mice [17, 18]. Supplementation of systemic GDF11 levels reversed functional impairments, restored genomic integrity in aged muscle stem cells, improved muscle functions and increased strength [17]. Moreover, treatment of old mice to restore GDF11 to youthful levels reversed age-related cardiac hypertrophy [16]. Several recent reports, however, have questioned these positive findings due to the cachectic effects of GDF11 when administered to mice [20–22, 32]. One possible reason for the controversy is that these initial studies relied on GDF11 serum or plasma levels, which are low and challenging to detect, because the use of anti-GDF11 antibodies often cross-react with myostatin (GDF8). In our study we attempted to circumvent the abovementioned technical issues by using commercial recombinant GDF11 protein, without dealing with the assessment of endogenous GDF11 levels and without antibodies that could potentially display cross-reactivity. Our data suggest that GDF11 supplementation in HSC (LX2) models impinges on ALK5/SMAD2/3/AKT dependent signaling pathways. These pro-fibrogenic effects *in vitro* were accompanied by the absence of modulation of steatosis upon administration of GDF11 for 1-2 weeks either to wild type mice or to genetically obese *ob/ob* mice, respectively. In fact, we found that GDF11 induced weight loss in both models without serious alterations of food intake, consistent with other studies [13, 23]. GDF11 has been shown to generate caloric-restriction like effects, with the adipose-tissue dependent secretion of adiponectin, which improves metabolic homeostasis [23]. However, adiponectin secretion is impaired and not affected by GDF11 treatment in leptin-deficient *ob/ob* mice [*data not shown* and [33]. Leptin might thus be required for GDF11-induced adiponectin secretion during obesity, and a significant correlation between circulating GDF11 and leptin levels has been found in obese patients [34]. RNA-Seq and bioinformatics analyses of GDF11-treated obese mice revealed a significant inhibition of genes belonging to the anti-fibrotic AHR, BRCA1-dependent DNA repair and HGF pathways [24–26]. Thus, the transcriptomic differences induced by GDF11 *in vivo* are suggestive of pleiotropic pro-fibrogenic effects. GDF11-treated HSCs became activated, as demonstrated by the upregulation of MMPs, actin and collagens. These *in vitro* findings were corroborated by the fact that GDF11 supplementation exacerbated NAFLD in 9-week-old *ob/ob* mice, which present extensive basal hepatic fat accumulation, inducing the appearance of periportal/perivenular fibrosis development. In the long term, *ob/ob* mice show metabolic, histological, and transcriptomic dysfunctions similar to human NASH, suggesting the great potential of this experimental model to discover novel drugs for NASH [35]. However, *ob/ob* mice develop spontaneous NASH, with hepatocellular ballooning and necroinflammatory foci at 20 weeks-of age. The transition from NAFLD to NASH in *ob/ob* mice depends on Toll-Like Receptor 4 (TLR4) expression [36]: GDF11 interacts with the TLR4/NF-kBp65 pathways in other inflammatory disorders [37, 38], and it was unclear if this occurs also in NAFLD/NASH. Our data demonstrate the absence of liver injury induced by GDF11 as well as a decrease in the number of liver Kupffer cells/macrophages, which normally attain a prominent role during liver inflammation. This is similar to what observed in the vessels of atherosclerotic ApoE^-/-^ mice administered with recombinant GDF11: a selective decrease in macrophages concomitant to an increase in collagen content [39]. We conclude that GDF11 may favor the first stages of NASH development, characterized by ECM deposition and HSC activation, without injury and inflammation. We cannot exclude that chronic/long term GDF11 administration might lead to more severe NASH, compared to acute/short term administration.

Translational relevance of our animal data, were confirmed by data obtained in a cohort of morbidly obese Italian individuals. Indeed, we found a strong positive correlation between hepatic GDF11 mRNA expression levels, NAS score and the mRNA levels of PPARγ, CPT1, SREBP1 and Col1A1 with the progression of the disease from NAFLD to NASH in our cohort. Moreover, consistently with data reported in German and in Chinese individuals [15], we showed an increased hepatic expression of GDF11 mRNA in the presence of fibrosis, mainly in mild fibrosis. Even though, the small sample size constrained our ability to provide a statistically significant correlation with all fibrosis stages, our results if validate in other cohorts could reinforce the relevance of GDF11 as therapeutic target to reduce NAFLD-associated liver damage. GDF11 has potent effects on metabolic and hepatic health. It is thus currently unclear, with respect to the regulation of GDF11 action, the impact of systemic *versus* local delivery, the implications of distinct interactions in diverse cellular contexts, whether recombinant and endogenous forms exert differential activity, and how concentration may influence GDF11 action. Given the current pharmaceutical investments to develop anti-aging strategies, including targeting GDF11 [40], it is of utmost importance to identify potential adverse effects at the population level. Our data suggest that GDF11 supplementation might not be completely safe as it triggers the first steps of fibrosis development, with HSC activation and liver ECM deposition. A deep understanding of the role of GDF11 in metabolically active tissues and immune cell types would likely open opportunities to develop new safe therapeutics for metabolic disorders.

## MATERIALS AND METHODS

### Patients - liver histology and gene expression

A total of 33 morbidly obese patients undergoing bariatric surgery were consecutively enrolled between January 2015 and December 2018 at the Bariatric Surgery Unit of the University Hospital of Palermo, Italy. The study was conducted in accordance with the Helsinki Declaration (1975). All participants provided written informed consent, and the University Hospital of Palermo Human Ethics Committee approved the study procedures. Before surgery, all patients were interviewed and examined during an outpatient visit. A standard questionnaire was used to establish the presence of cardiovascular, metabolic or liver diseases, use of potentially hepatotoxic drugs and alcohol consumption. In all subjects, height (m), weight (kg), and body mass index (BMI) were measured. Blood pressure was evaluated and hypertension was diagnosed according to the WHO/ISH criteria (systolic blood pressure ≥140 mm Hg and/or diastolic blood pressure ≥90 mm Hg). Before surgery, fasting blood samples were obtained to assay glucose, total cholesterol, triglycerides and HDL, alanine aminotransferase (ALT), aspartate aminotransferase (AST), gamma-glutamyl transferase (GGT), total bilirubin and insulin levels. The insulin resistance (IR) index was calculated using the homeostasis assessment model (HOMA-IR). Patients who had elevated transaminase values were tested for HBsAg, HCV, anti-nuclear antibodies (ANA), anti-mitochondrial antibodies (AMA), anti-smooth muscle antibodies (ASMA), and liver kidney microsomal antibody type 1 (LKM1) antibodies. Patients who had an etiologically well-defined liver disease were excluded from the study. Wedge or core liver biopsies were obtained during surgery. One part of the liver biopsy was immediately fixed in formalin for histological assessment and another part was snap frozen in liquid nitrogen and stored a -80° C for molecular analyses. H&E and picrosirius red staining were performed in each case. A diagnosis was made using the NAFLD activity scoring (NAS) system [41], and the fibrosis score, by one expert pathologist (D.C.) who was unaware of patient identity and history.

Total RNA was extracted from the frozen liver biopsies using TRIzol reagent (Invitrogen), following the manufacturer’s instructions. Total normal liver RNAs were obtained from five donor pools (Biochain, Newark, CA) and from four donor pools (Takara BioUSA, Mountain View, CA, USA). Then, 1.5 μg of total RNA were used for reverse transcription to generate cDNA and real-time PCR was performed. The levels of mRNA were evaluated using specific QuantiTect Primer Assays (QIAGEN) for GDF11 (QT00101808), (QT00082236), PPARα (QT00017451), PPARγ (QT00029841), SREBP1 (QT00036897), FASN (QT00014588) and Col1A (QT00037793). Each sample was analyzed in triplicate, and gene expression values were normalized to the values of 18S ribosomal RNA (QT00199367) and of RPL32 (QT00046088), which were both used as internal controls, according to the GeNorm method [42].

### Cell culture

The human stellate cell line LX2 was purchased from American Type Culture Collection (ATCC, Manassas, VA) and was cultured in DMEM (1X) supplemented with 10% fetal bovine serum (FBS) (Sigma-Aldrich, Czech Republic), 15 mM Hepes buffer (Biowest, France), Glutamine, 1% penicillin/streptomycin solution and 100 μg/ml Normocin. The medium was routinely changed every 2 days and the cells were sub-cultured using TrypLE Express (Gibco, Ireland) when reaching 90% confluence. For experiments, cells at 60% confluence were treated with 25-50-100 ng/ml recombinant GDF11 (PeproTech, NJ, US) and/or 25 μM Repsox inhibitor and human Insulin solution 100nM/ml (I9278, Sigma-Aldrich). When GDF11 and Repsox inhibitor were used in combination, the cells were pretreated with 25 μM Repsox for 4h before GDF11 was added and co-cultivated for a further 24 or 48h.

### Microscopy and immunofluorescence in cell lines

Immunofluorescence in cells were performed as previously described [43]. Briefly, treated cells that were seeded on coverslips (12 mm) were fixed using 4% buffered paraformaldehyde solution in PBS for 10 min, washed 3-5 times with PBS and permeabilized with 0.2% Triton-X-100 in PBS for 10 min. After three further washes with PBS for 3 min each, the cells were incubated with blocking solution containing 3% bovine serum albumin (BSA) in PBS for at least 1 h at room temperature (RT). Fixed and permeabilized samples were incubated overnight at 4° C with primary rabbit anti-SMAD2/3 (1:500; Cell Signaling Technology, MA, US), which was diluted in blocking solution. After 3-5 washes with PBS, the samples were incubated with secondary antibody goat anti-rabbit AlexaFluor488 (1:1000, Invitrogen, CA, US) at RT for 1h. After incubation, the slides were washed three times with PBS. F-actin was then detected using AlexaFluor555-labeled phalloidin dye (ThermoFisher Scientific, MA, US) to visualize the cell borders. Cell nuclei were counterstained with DAPI (1ug/ml) solution. All samples were then mounted in Mowiol hardening media and images were captured using an Axio scan Z.1 (Zeiss) equipped with a Hamamatsu ORCA-Flash 4.0 camera and ImageJ software analysis program (NIH Image, Bethesda, MD) was used to evaluate all immunofluorescence images. Nuclear/cytoplasmic ratio of Smad2/3 was evaluated in three, blindly chosen fields-of-view from each sample group at 400x magnification.

### Gene expression assay

Genomic RNA was isolated by column separation using an RNeasy mini Kit (Qiagen, Germany), according to manufacturer's instructions. Three biological replicates of LX2 cells were prepared for each treatment group (CTL, PBS; GDF11 100 ng/ml; and/or FFA 100uM). After quantification on NanoDrop 1000 spectrophotometer (ThermoFisher Scientific), 1μg of total isolated RNA was used to prepare cDNA using a High-Capacity cDNA Reverse Transcription Kit (ThermoFisher Scientific). Real Time-PCR was performed in two or three technical replicates using a StepOnePlus™ Real-Time PCR System (Applied Biosystems, Darmstadt, Germany) and SYBR™ Select Master Mix (ThermoFisher Scientific). The PCR reaction was held in 10ul volume and 250 ng cDNA per well was used as the input quantity. The primer sequences used in this study are listed in Supplementary Table 4.

### Immunoblotting analyses

Protein extraction and immunoblotting analyses were performed as previously described [44, 45]. Antibodies used in this study: Cell Signaling Technology (MA, USA) - rabbit anti-SMAD2/3 primary antibody (1:500), rabbit anti-Phospho-Smad2 (Ser465/467, 1:500) rabbit anti-Akt (1:1000), rabbit anti-Phospho-Akt (Ser473) (1:1000), rabbit anti Vimentin (1:1000), Abcam (UK) – mouse anti-β-Actin (1:1000), rabbit anti Collagene I (1:1000), rabbit anti αSMA (1:1000), ThermoFischer Scientific (CA, USA) - mouse IgG1 GAPDH monoclonal HRP conjugated antibody (1:2000).

### Mice models

16-18 months old C57BL/6J wild type mice livers were obtained from (23). The use of *ob/ob* mice complied with the institutional and European legislation concerning vivisection, the use of genetically modified organisms, animal care and welfare (European Directive 2010/63/UE adopted by the European Parliament and the Council of the EU on September 22, 2010). The granted experimental protocol n°516/2018-PR was approved by the University of Brescia Institutional Animal Care Committee (Brescia, Italy) and was conducted in accordance with national and European regulations. *Ob/ob* mouse lines were maintained on a C57BL/J6 background within the University of Brescia animal facility (Brescia, Italy), in temperature controlled rooms under a 12h light/dark cycle, in conventional cages with enriched environment and standard diet. Mice had access to food and water ad libitum.

### Histological and immunofluorescence analyses

Samples of murine liver tissue were embedded and snap frozen in Tissue Freezing Media (Leica Microsystems, Wentzler, Germany) and were cut to 7μm at -20° C with a cryostat (Leica Microsystems). The slides were then processed by hematoxylin and eosin (H&E) staining and Masson´s trichrome staining for histological evaluation, as described previously [45–47]. Briefly, Infiltration of the liver with fat/steatosis was assessed using the ImageJ software analysis program on H&E-stained sections by counting 200 randomly chosen lipid droplets per sample/mouse (n=6 mice per group, GDF11/CTL groups) and selecting their diameter (um) and area (um^2^) at 400x magnification. The morphometrical analyses were performed by two different observers blinded to the study conditions, using Image Pro Premier 9.1 (MediaCybernetics Inc., OR, USA). Diagnostic classification of NAFLD was performed by applying a semi-quantitative scoring system that grouped histological features into broad categories (steatosis, hepatocellular injury, portal inflammation, fibrosis and miscellaneous features), as previously described [41]. Masson‘s trichrome staining was used to identify liver fibrosis. The extent of fibrosis was then evaluated using Image Pro Premier 9.1 (MediaCybernetics Inc., OR, USA) that scanned 20 randomly chosen liver fields per experimental animal (n=6 mice per group) at 400x magnification. Liver fibrosis was evaluated as the % of the total imaged area from the respective analyzed field. Immunofluorescence detection in mouse liver tissues was used for the detection of stellate cell activation (αSMA/ACTA2) and liver macrophage infiltration (F4/80; CD11b). Slides with 10μm cut samples of embedded snap frozen murine liver tissues were blocked in M.O.M. (Mouse on Mouse) Blocking Reagent (Vector Laboratories Inc., CA, USA) for 1h. Primary antibodies from Abcam: rat anti-F4/80 (1:200; ab6640); rabbit anti-αSMA (1:100; ab5694) and from Biolegend (CA, USA): biotin-rat anti-CD11b (1:150, 101204) were diluted in DAKO antibody diluent (Agilent technologies) and incubated overnight in humid chamber. After three consecutive washes with PBS, mix of secondary antibodies (1:500): Streptavidin-AF647 (Biolegend 405237) Goat anti-Rat IgG AF546 (A11081) and/or Goat anti-Rat IgG AF647 (A21247) was applied and incubated for 1h. After washing three times with PBS, slides were counterstained with DAPI (1ug/ml) solution and mounted in Mowiol hardening media. Images were captured using an Axio scan Z.1 equipped with a Hamamatsu ORCA-Flash 4.0 camera and ImageJ software analysis program was used to evaluate all immunofluorescence images. Macrophage infiltration or αSMA abundance in mouse liver samples was evaluated as % of positive area per frame at least in fifteen blindly chosen fields-of-view from each sample at 200x magnification.

To evaluate the degree of apoptosis rate, Click-iT TUNEL AF647 Imaging Assay (C10247, Thermofisher) was used according to manufacturer’s instruction. Slides were then scanned using Axio scan Z.1 and image analysis was done by ImageJ software, where, at least six blindly chosen fields-of-view were evaluated.

### RNA-Sequencing (RNA-Seq)

Total RNA was extracted from three biological replicates of control and GDF11 treated livers from ob/ob mice with TRIzol Reagent (Invitrogen, CA, US) and column separation using a DNeasy Blood and Tissue Kit (Qiagen, Germany), according to manufacturer's instructions. RNA integrity was assessed using Agilent RNA 6000 Nano Kit and Agilent 2100 Bioanalyzer (both Agilent Technologies, CA, US). Indexed libraries were prepared from 250 ng purified RNA using a NEB Next Ultra II Directional RNA Library Prep Kit (Illumina, UK), according to the manufacturer's instructions. The libraries were pooled in equimolar amounts, subjected to cluster generation and sequenced on an Illumina MiSeq System (Illumina) in a 1x75 format. Quality control was performed by FastQC and MultiQC. Raw counts were transformed by the regularized logarithm (rlog) transformation function. Raw sequences pertaining the RNA-Seq experiment of control and GDF11 treated ob/ob mice were trimmed by Trimmomatic ver. 0.35, because of systematic sequencing errors in the first 11 nucleotides, quasi-mapped against the GRCm38 reference mouse genome and quantified by Salmon ver. 1.0.0, considering the Gencode M23 transcript definition and GFF3 genome annotation. In both experiments, differential expression analysis was performed using the DESeq2 R package. Pathway enrichment analysis wasperformed by Ingenuity Pathway Analysis (IPA).

### Statistical analyses

Comparisons between groups were made using the parametric Student's t-test, if the Normal distribution was proven, or the non-parametric Mann–Whitney U-test was used instead. To determine statistical significance between more than two groups, a parametric One-Way ANOVA was used when the data had a normal distribution, or otherwise a non-parametric Kruskal-Wallis test, as appropriate. Statistical analyses were performed in GraphPad Prism Software (version 7.00 for Windows; GraphPad Inc., CA, USA). Functional and pathway enrichment analyses on differentially expressed lipid metabolism genes were made using the Ingenuity Pathway Analysis (IPA, spring 2018 release, QIAGEN Inc., https://www.qiagenbioinformatics.com/products/ingenuity-pathway-analysis) software package. Genes were considered differentially expressed between groups if their expression values significantly differed by >2 fold with a P ≤0.05. Correlations between GDF11 gene expression levels and other genes, and between GDF11 gene expression levels and patients clinical data were determined by Pearson χ2 test for categorical variables (variables with limited or fixed, number of possible values). Independent experiments were carried out at least 2-3 times with 2-3 technical replicates. The data are expressed as the means ± SD (unless indicated otherwise). Differences were considered statistically significant at p<0.05 (*), p<0.01 (**) and p<0.001 (***).

All authors had access to the study data and had reviewed and approved the final manuscript.

## Supplementary Material

Supplementary Figures

Supplementary Tables

Supplementary File 1
