# Supplementary material for: GDF11 induces mild hepatic fibrosis independent of metabolic health
Source: Aging (Albany NY). 2020 Oct 28;12(20):20024–46. doi: 10.18632/aging.104182 (PMC7655202; doi:10.18632/aging.104182)
Supplement: Supplementary Figures [file aging-12-104182-s001..pdf]

SUPPLEMENTARY FIGURES

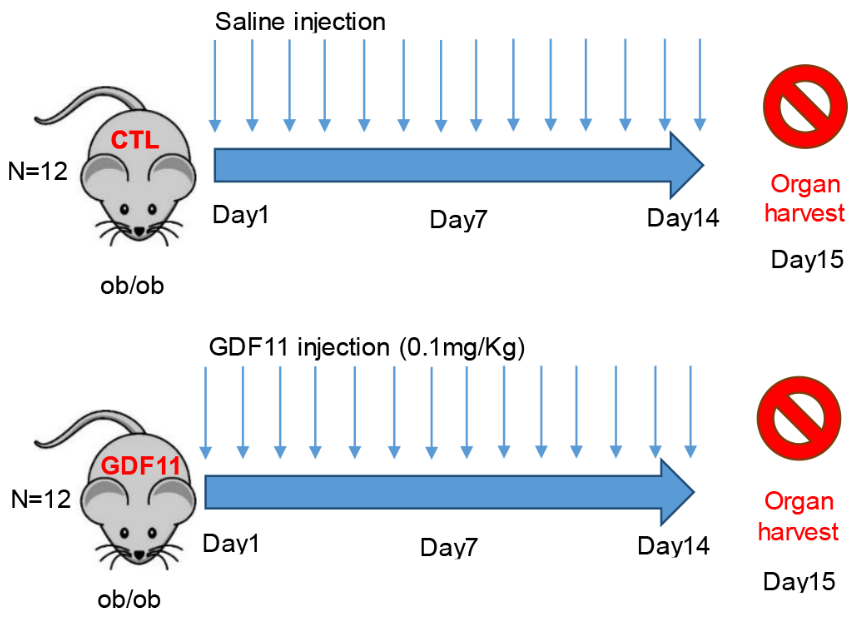

Supplementary Figure 1. I*n vivo* experimental setup using *ob/ob* mice. Two experimental groups were injected daily (14 days) with either GDF11 (0,1 mg/kg) or saline (n=12 per group).

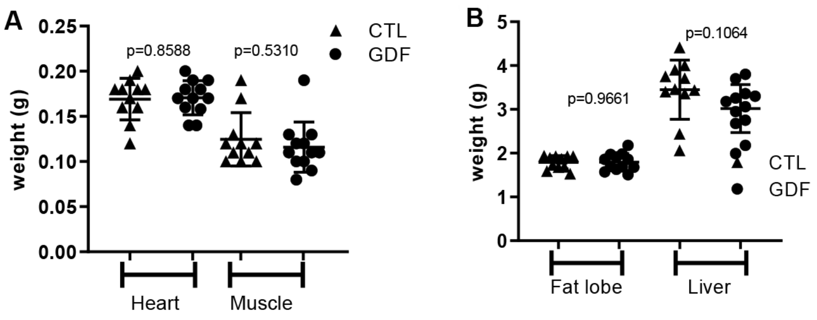

Supplementary Figure 2. Organ weight in *ob/ob* BL/6 mice. Two experimental groups were injected daily (14 days) with either GDF11 (0.1 mg/kg) or saline (n=12 per group). (**A**) Graph showing average weight of heart and muscle (quadriceps). (**B**) Graph showing average weight of abdominal fat lobe and liver. No significance was observed between treatment groups (Mann-Whitney U-test).

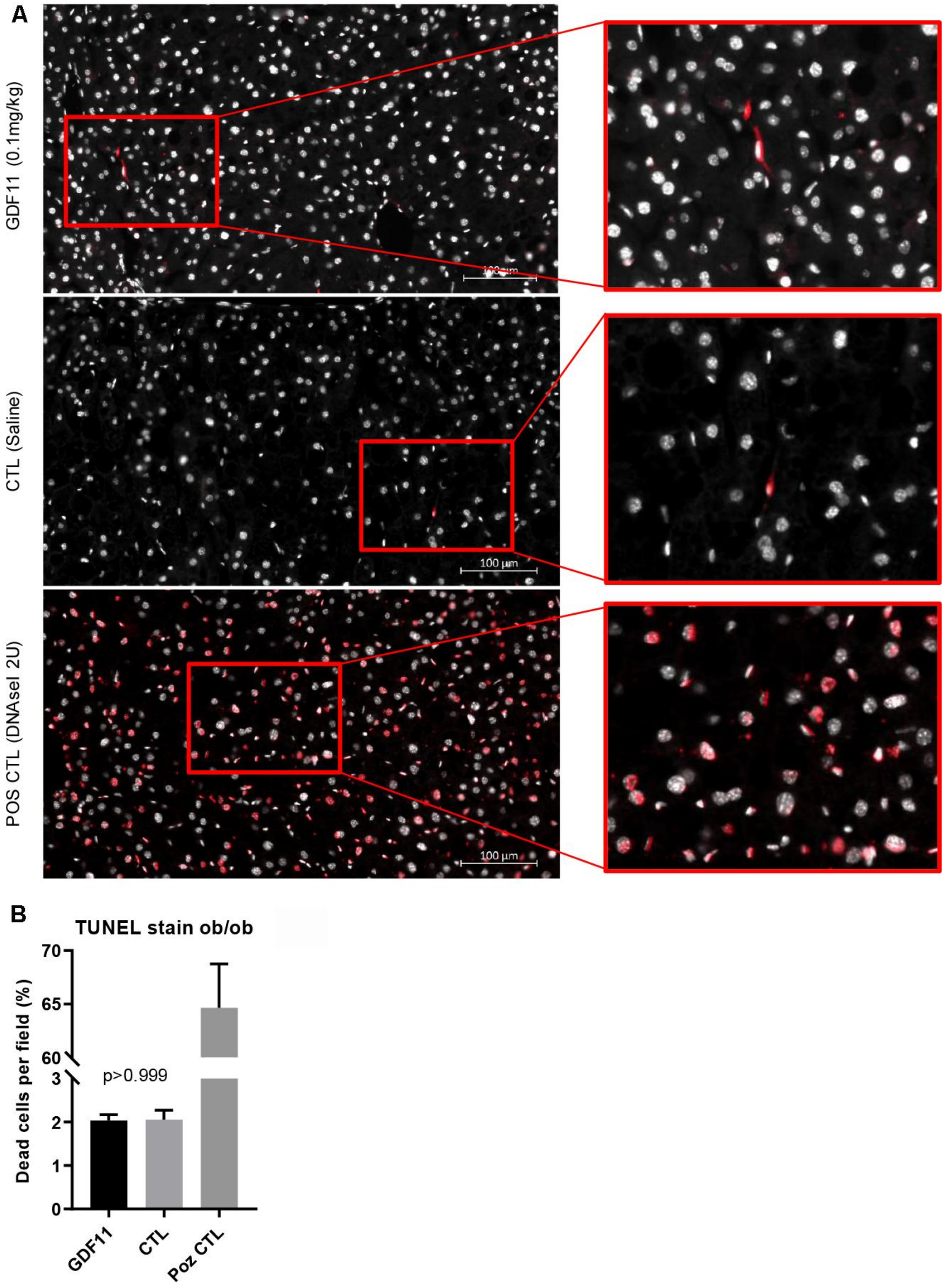

Supplementary Figure 3. TUNEL staining in *ob/ob* liver samples. (**A**) Two experimental groups were injected daily (14 days) with either GDF11 (0,1 mg/kg) or saline (n=6 per group). As positive controls were used samples from both groups treated with DNAse I (2U in 70µl) to induce breaks in DNA structure. (**B**) Graph showing quantification of TUNEL stain in *ob/ob* liver samples (n=6 per group, n=2 per Pos.CTL). (Mann-Whitney U-test).

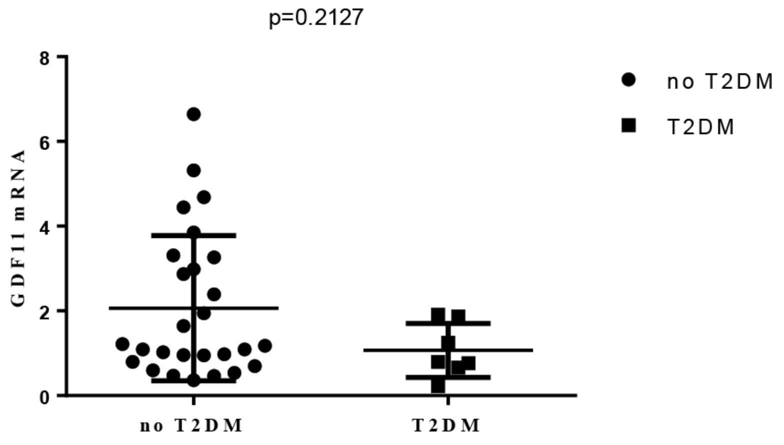

Supplementary Figure 4. Plot showing GDF11 mRNA levels in patients with or without type II diabetes (T2DM) (Mann-Whitney U-test).
