# Supplementary material for: GDF11 induces mild hepatic fibrosis independent of metabolic health
Source: Aging (Albany NY). 2020 Oct 28;12(20):20024–46. doi: 10.18632/aging.104182 (PMC7655202; doi:10.18632/aging.104182)
Supplement: Supplementary Tables [file aging-12-104182-s002..pdf]

SUPPLEMENTARY TABLES

Supplementary Table 1. Anthropometric, clinical and pathological features of 33 morbidly obese patients (BMI average 45.02 ± 6.41).

Clinical/pathologic characteristics of morbidly obese patients (n=33)	
Age (years)	37 ± 10.37
Gender (M/F)	9/25
Weight (kg)	123.74 ± 18.71
BMI (kg/m^2)	45.02 ± 6.41
HDL - cholesterol (mg/dL)	52.03 ± 15.07
LDL - cholesterol (mg/dL)	116.05 ± 30.47
Total cholesterol (mg/dL)	192.28 ± 31.29
Triglycerides (mg/dL)	120.97 ± 60.27
ALT (IU/L)	38.91 ± 29.13
AST (IU/L)	25.37 ± 15.48
GGT (IU/L)	52.37 ± 144.46
Total Bilirubin (mg/dL)	0.44 ± 0.2
Blood glucose (mg/dL)	96.35 ± 32.62
Insulin (mIU/L)	28.93 ± 24.31
HOMA-IR	7.81 ± 8.38
Hb1Ac (%)	5.71 ± 0.49
Kleiner steatosis grade	
0	4
1	11
2	6
3	12
Kleiner lobular inflammation grade	
0	4
1	18
2	9
3	2
Kleiner ballooning score	
0	12
1	12
2	9

Supplementary Table 2. Pearson's correlation between GDF11 mRNA and clinical/pathological characteristics of morbidly obese patients (n=33).

	GDF11 mRNA	
	**r**	**p**
**Age**	0.1203	0.4978
**Gender**	-0.023	0.90
**BMI**	0.094	0.5967
**ALT**	0.2745	0.1221
**AST**	0.017	0.9279
**GGT**	0.0366	0.8423
**Total Bilirubin**	0.084	0.639
**HDL**	-0.1740	0.366
**LDL**	0.093	0.631
**Total cholesterol**	-0.0273	0.888
**Triglycerides**	-0.088	0.645
**Blood glucose**	0.2981	0.08
**Insulin**	0.1650	0.401
**HOMA-IR**	0.3174	0.1141
**HbA1c**	0.1231	0.5247

Kleiner Score (0-8)	**0.3664**	**0.0360**
Steatosis grade (0-3)	0.2946	0.1077
Inflammation score (0-3)	0.2778	0.1303
Ballooning score (0-2)	0.3450	0.057

Supplementary Table 3. Pearson's correlation between GDF11 mRNA and glucose/insulin characteristics in the obese patients with either NASH (n=13) or NAFLD (n=20).

**NAFLD**	**GDF11 mRNA vs. Blood glucose**	**GDF11 mRNA vs. Insulin**	**GDF11 mRNA vs. HOMA-IR**
Pearson r	-0.4711	-0.3572	-0.3437
P value	0.036	0.1744	0.2289
P value summary	*	ns	ns
Significant? (alpha = 0.05)	Yes	No	No
**NASH**	**GDF11 mRNA vs. Blood glucose**	**GDF11 mRNA vs. Insulin**	**GDF11 mRNA vs. HOMA-IR**
Pearson r	-0.0949	-0.2043	-0.1818
P value	0.7577	0.5242	0.5717
P value summary	ns	ns	ns
Significant? (alpha = 0.05)	No	No	No

Supplementary Table 4. The primer sequences used in this study.

	**Gene**		**Sequence (5′-3′)**
Homo sapiens	VIMENTIN	F	AGTCCACTGAGTACCGGAGAC
		R	CATTTCACGCATCTGGCGTTC
	COL5A1	F	TACAACGAGCAGGGTATCCAG
		R	ACTTGCCATCTGACAGGTTGA
	MMP2	F	TACAGGATCATTGGCTACACACC
		R	GGTCACATCGCTCCAGACT
	COL1A1	F	GTGCGATGACGTGATCTGTGA
		R	CGGTGGTTTCTTGGTCGGT
	ACTA2	F	AAAAGACAGCTACGTGGGTGA
		R	GCCATGTTCTATCGGGTACTTC
	TIMP1	F	ACCACCTTATACCAGCGTTATGA
		R	GGTGTAGACGAACCGGATGTC
	GDF11	F	CCACCACCGAGACCGTCATT
		R	GAGGGCTGCCATCTGTCTGT
	GAPDH	F	GGTGCGTGCCCAGTTGA
		R	TACTTTCTCCCCGCTTTTT
	ACTB	F	CATGTACGTTGCTATCCAGGC
		R	CTCCTTAATGTCACGCACGAT
